# Evaluation of methods for assigning causes of death from verbal autopsies in India

**DOI:** 10.3389/fdata.2023.1197471

**Published:** 2023-08-24

**Authors:** Sudhir K. Benara, Saurabh Sharma, Atul Juneja, Saritha Nair, B. K. Gulati, Kh. Jitenkumar Singh, Lucky Singh, Ved Prakash Yadav, Chalapati Rao, M. Vishnu Vardhana Rao

**Affiliations:** ^1^Indian Council of Medical Research-National Institute of Medical Statistics, New Delhi, India; ^2^World Health Organization, New Delhi, India; ^3^College of Health and Medicine, Australian National University, Canberra, ACT, Australia

**Keywords:** cause of death, computer-coded verbal autopsy (CCVA), physician-coded verbal autopsy (PCVA), verbal autopsy, India

## Abstract

**Background:**

Physician-coded verbal autopsy (PCVA) is the most widely used method to determine causes of death (COD) in countries where medical certification of death is low. Computer-coded verbal autopsy (CCVA), an alternative method to PCVA for assigning the COD is considered to be efficient and cost-effective. However, the performance of CCVA as compared to PCVA is yet to be established in the Indian context.

**Methods:**

We evaluated the performance of PCVA and three CCVA methods i.e., InterVA 5, InSilico, and Tariff 2.0 on verbal autopsies done using the WHO 2016 VA tool on 2,120 reference standard cases developed from five tertiary care hospitals of Delhi. PCVA methodology involved dual independent review with adjudication, where required. Metrics to assess performance were Cause Specific Mortality Fraction (CSMF), sensitivity, positive predictive value (PPV), CSMF Accuracy, and Kappa statistic.

**Results:**

In terms of the measures of the overall performance of COD assignment methods, for CSMF Accuracy, the PCVA method achieved the highest score of 0.79, followed by 0.67 for Tariff_2.0, 0.66 for Inter-VA and 0.62 for InSilicoVA. The PCVA method also achieved the highest agreement (57%) and Kappa scores (0.54). The PCVA method showed the highest sensitivity for 15 out of 20 causes of death.

**Conclusion:**

Our study found that the PCVA method had the best performance out of all the four COD assignment methods that were tested in our study sample. In order to improve the performance of CCVA methods, multicentric studies with larger sample sizes need to be conducted using the WHO VA tool.

## 1. Introduction

Verbal autopsy (VA) is a method of ascertaining cause of death (COD) from information on signs/symptoms and circumstances preceding death through interviewing the deceased's caretakers (Registrar General of India, 2016). VA has conventionally been used as a research tool for longitudinal population studies, intervention research and epidemiological studies. VA has also been used to collect COD statistics at the population level in some countries to provide cause-specific mortality data for priority setting for planning and policy formulation (Mahapatra and Chalapati Rao, 2001; Registrar General of India, 2018). In India, where the availability of a medically certified COD is only 23% (Jha et al., 2006; Soleman et al., 2006; Fottrell and Byass, 2010), data collection for COD statistics will continue to rely on VA methods (Bauni et al., 2011; Murray et al., 2011a, 2014). These methods have been regularly used by India's Sample Registration System (SRS) for mortality measurement since 2001. In the SRS, VA questionnaires are administered to the family of the deceased by trained interviewers, and completed forms are reviewed by a team of physicians who assign and code the COD. In recent times, several computerized diagnostic programs have been developed for automated assignment of causes of death, which has the potential to improve efficiency in data processing and timely compilation of required information. Currently, there are three commonly used computerized VA COD assignment programs available in the public domain, which are the InterVA5, InSilicoVA, and Tariff programs (Nichols et al., 2018). There is a need to establish the accuracy of these programs for computerized coding of VA (CCVA) in comparison with the physician coding of VA (PCVA) approach, to guide decisions on the optimal VA methodology for India. This article describes the methods and results from a field study to measure and compare the validity of different VA diagnostic approaches, in order to establish the utility of the resultant information on causes of death.

For validation, there is a need for a reference standard underlying cause of death (UCOD) for comparison with the underlying cause derived from PCVA or any of the CCVA methods. For the same death. The reference UCOD can be derived from the pathological autopsy, which is considered as a “gold standard,” or from clinical records which are considered as the next best alternative (Murray et al., 2011b). From a practical standpoint, a hospital diagnosis of underlying COD which is based on defined laboratory and clinical criteria, are the most viable option for validating VAs (Landis and Koch, 1977). VA validation studies conducted in several countries have used the hospital medical records (MRs) of inpatient deaths as reference diagnoses (Quigley et al., 1999; Rao et al., 2005; Murray et al., 2011b). Previous studies have attempted to validate and compare the performance across different CCVA and PCVA methods, but these efforts have been hampered by variations in the design and content of VA questionnaires used for data collection by each diagnostic method, which have resulted in inconsistencies in findings of comparative validation (Fottrell and Byass, 2010). This study uses a recently developed set of international VA standards for data collection, which enables simultaneous direct analyses by three commonly used CCVA methods as well as the PCVA method, which will ensure unbiased interpretation of the comparative performance of different methods.

## 2. Materials and methods

### 2.1. Study design and setting

A cross-sectional study was designed to validate the causes of a sample of deaths that occurred in five selected tertiary hospitals in New Delhi. These hospitals were selected because of their high volume of patients as well as the availability of high-level diagnostic services to establish reference diagnoses for validation. Also, these hospitals covered a wide range of services for communicable diseases, maternal and child health, internal medicine, and surgery and were expected to yield cases across the spectrum of diseases of interest for diagnostic validation in this study.

For each selected hospital, essential details of identity and address of all deaths that occurred during the period from 01st July 2016 to 30th June 2017 were obtained from the city municipal corporation. Cases were screened for inclusion in the sampling frame of the study if they met the initial eligibility criteria of the deceased being an Indian national, whose place of residence was within a 250 km radius of Delhi, and for whom a detailed address was noted on the medical record. The study involved data collection in two sequential steps, the first being to review clinical records in hospitals and establish cases with high-quality clinical evidence to serve as reference standards, and the second step in completing a household VA interview to arrive at the VA diagnosis (by PCVA/CCVA) which would be compared with the reference standard for validation.

### 2.2. Sampling plan

The key objective of the study was to establish the most accurate approach for assigning the COD to Verbal Autopsy tool. The parameter of accuracy or estimating the sample size was chosen to be the sensitivity of a COD assignment method in correctly assigning diagnoses from a specific cause of interest when compared to the set of cases with reference standard diagnoses from that cause. Based on the literature review, it was estimated that to establish a sensitivity of 70% with an absolute margin of 5–6% with a confidence of 95%, an estimated sample size of 250 cases with reference diagnoses for the cause of interest would be required. Since the study proposed to validate 20 specified causes of death, a total sample of 5,000 cases was required, with equal distribution across these 20 causes of interest. Considering the potential for attrition and loss to follow-up due to various reasons, the size of this primary sample was inflated by 40% to 7,000 cases.

### 2.3. Data collection to establish reference diagnoses

In each hospital, a nominated team member prepared medical record files during the study time period by de-identification, assignment of the study case number, and removal of the medical certificate of COD, which were then sent for blind physician review. A team of trained physicians in medical certification of COD and basic rules for selection of the underlying COD as per ICD (International Classification of Diseases) procedures reviewed the case records in each hospital. All cases that met the criterion for the confirmed diagnosis and were from the list of 20 selected causes of interest were selected into the study sample. Confirmatory evidence comprised of either an appropriate laboratory test, imaging investigation, surgical notes or documented clinical history and observations suggestive of the diagnosis of interest, similar to criteria used in other studies (Murray et al., 2014). Each case was then assigned the specific code for the underlying cause from the WHO modified VA list (World Health Organization, 2017a), which was categorized as the first physician (P1) diagnosis for the case. Subsequently, all records with P1 diagnoses were further subjected to an independent review by a second physician, who assigned a diagnosis and code for the underlying cause, termed as the P2 diagnosis. All cases with matched P1 and P2 diagnoses were included as cases with reference diagnosis for validation. Cases with unmatched P1 and P2 diagnosis were then subjected to an additional independent review (P3), and if the P3 matched either P1 or P2, the case was included in the validation sample. Cases that were not from the 20 selected cases, or did not have adequate medical records with confirmatory evidence, or for which there was no agreement between any two of the three independent reviews were excluded from the sample. During the entire review process, reviewing doctors provided informed consent to keep all identities and case characteristics confidential.

### 2.4. Verbal autopsy data collection

International standard VA questionnaires developed by the World Health Organization in 2016 were used for this study. These questionnaires include a list of structured items covering the medical history, clinical symptoms and signs, and associated circumstances pertaining to the terminal illness of the deceased. These structured items included all the key variables required for each of the three CCVA diagnostic programs, hence ensuring compatibility for direct comparisons. In addition, the questionnaires also include a free text section for interviewers to record an open narrative from the respondents about their version of the illness and the terminal events of the deceased in their own words. This open narrative section is referred to by physicians in their process of deriving the underlying COD for each case. The questionnaires were translated into Hindi and also back translated to check for the quality and accuracy of the translation. All materials (questionnaires, table of indicators interviewer manual) are available for download from the WHO Verbal autopsy website (World Health Organization, 2017b).

A team of 30 project assistants with graduate/qualifications in Social Work was recruited as VA interviewers for this study. A training manual adapted from the WHO interviewer manual using local language (Hindi) was developed for the training purpose. A 10-day training program of the field investigators was conducted which included 6 days of in-house training on the VA tool followed by 4-day field level training. The training was imparted by physicians having prior field experience in conducting VA and analyzing verbal autopsy data. They underwent a training program that included classroom sessions on VA methodology, detailed instruction and focus group discussions on questionnaire content and interviewing skills, VA ethics, as well as field practice interviews with feedback sessions. The sessions focused on highlighting the concept, importance and purpose of VA along with an in-depth understanding about the procedures, principles and communication techniques for conducting VA interviews. The in-house training also included mock role plays along with dedicated sessions for identification of signs and symptoms related to common illnesses. The practical sessions were facilitated by several of the co-authors on the manuscript, as well as other technical officers from the institute with previous experience in conducting VA studies. VA data was collected using tablet computer devices, for which interviewers were also familiarized with their use, which included instructions on program control, troubleshooting, and data storage and transmission. The cases were allotted to interviewer teams according to geographical clusters within suburban areas of Delhi. The project management team provided on-site supervision and troubleshooting tips to improve interviewing skills, monitored field progress and data quality, and arranged data storage.

The study protocol and all procedures were approved by the ICMR-NIMS- Institutional Ethical Committee and approved by the ICMR-NIMS Scientific Advisory Committee.

### 2.5. Data management and processing

Electronic data from all VAs were merged into a single data file which was cleaned and prepared for further analysis. The OpenVA package was used for analysis with the interVA-5 and InSilico CCVA diagnostic programs, and the Smart VA application was used for the Tariff method (Thomas, 2018). Each CCVA program generates a single probable COD for each case, which was used for subsequent validation and comparative analyses against the reference underlying COD from the hospital record for the same case. For the PCVA diagnosis, the electronic VA file for each case was merged with its open narrative to create a single electronic document for physician review. A team of 11 physicians was trained for PCVA, including the concepts and rules for selection of the underlying COD, according to the International Classification of Diseases and Health-Related Problems, Tenth Revision (ICD-10). Each case was reviewed by two physicians (P1 and P2) with proper blinding procedures. Subsequently, the same process for assignment of COD code as for the establishment of the reference standard cases from hospitals was followed, in terms of matching of COD assignment between two independent physicians (P1 and P2) and review by a third physician (P3) where required. All cases with matched underlying causes were considered as the final PCVA cases for VA validation analysis. Cases for which there was no matching (P1≠P2≠P3) were discarded from the analysis.

### 2.6. Analysis

A range of statistical indicators were used to measure and compare the accuracy of different techniques (PCVA and CCVA) for assigning causes of death. For accuracy of individual causes, the indicators used are the Percentage Relative Difference between proportions from the reference standard cases and the test method cases, as well as Sensitivity and Positive Predictive Values. The overall accuracy of a COD diagnostic method across all causes in comparison with the reference standards was evaluated using three indicators.

i. **Cause-Specific Mortality Fraction Accuracy (CSMF Accuracy)** is defined as the average value of differences between CSMFs for each cause by a particular COD assignment method and the CSMF for the same causes in the reference dataset (Murray et al., 2011b);
ii. **The agreement** is the proportion of the sum of positive agreements between a COD assignment method and the reference diagnoses across all COD, out of the total study sample.
iii. **Kappa statistics** (often simply called Kappa) is a measure of agreement between the two independent ratings for the same observation (Landis and Koch, 1977). Kappa Statistics can be interpreted according to different grades of agreement poor (< 0.0), slight (0.00–020), fair (0.21–0.40), moderate (0.41–0.60), substantial (0.61–0.80), almost perfect fair (0.81–1.00).
iv. **Cross tabulations** of causes from the reference dataset and each COD assignment method were also analyzed to review the misclassification of patterns and to understand the overall plausibility of diagnoses from the COD assignment method.

## 3. Results

**Figure 1:**
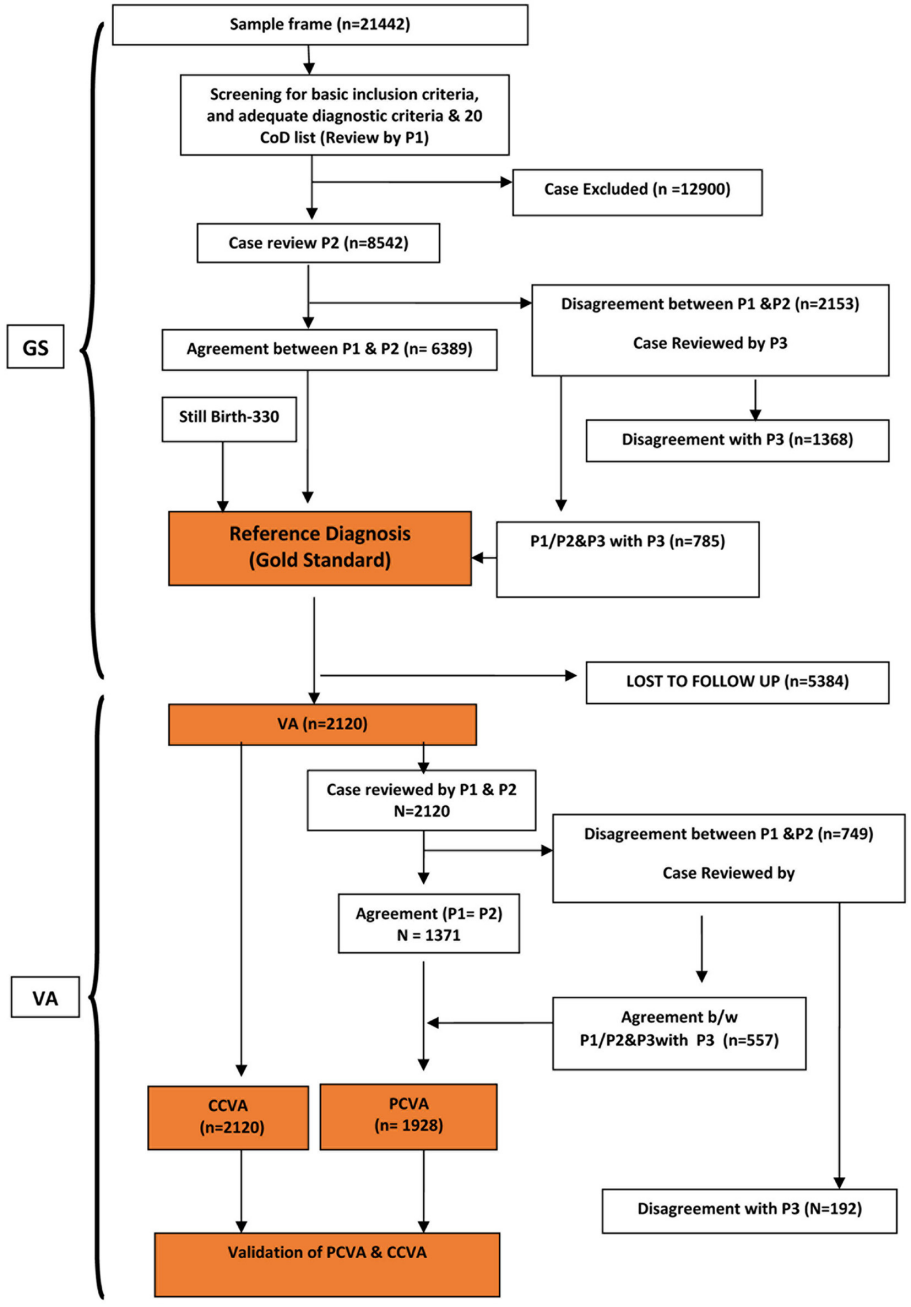
Flow chart depicting data collection across different components of the validation study.

Figure 1 shows the flow diagram of procedures for recruiting cases into the validation study sample. From a sampling frame comprising 21,442 deaths that had occurred in the five hospitals during the study reference period (2016–2017), a total of 8,542 cases met the initial eligibility criteria. Of these, 7,504 cases were from the 20 causes of interest for this study and had sufficient diagnostic evidence to serve as reference cases for validation. VA interviews were attempted for only 5,384 cases owing to logistical and time constraints and completed in 2,120 cases, yielding a field success rate of 39%. The majority of the 3,264 cases lost to field follow-up were due to inaccurate address records on case files (84%), and migration of the deceased's family (9%).

**Figure 2:**
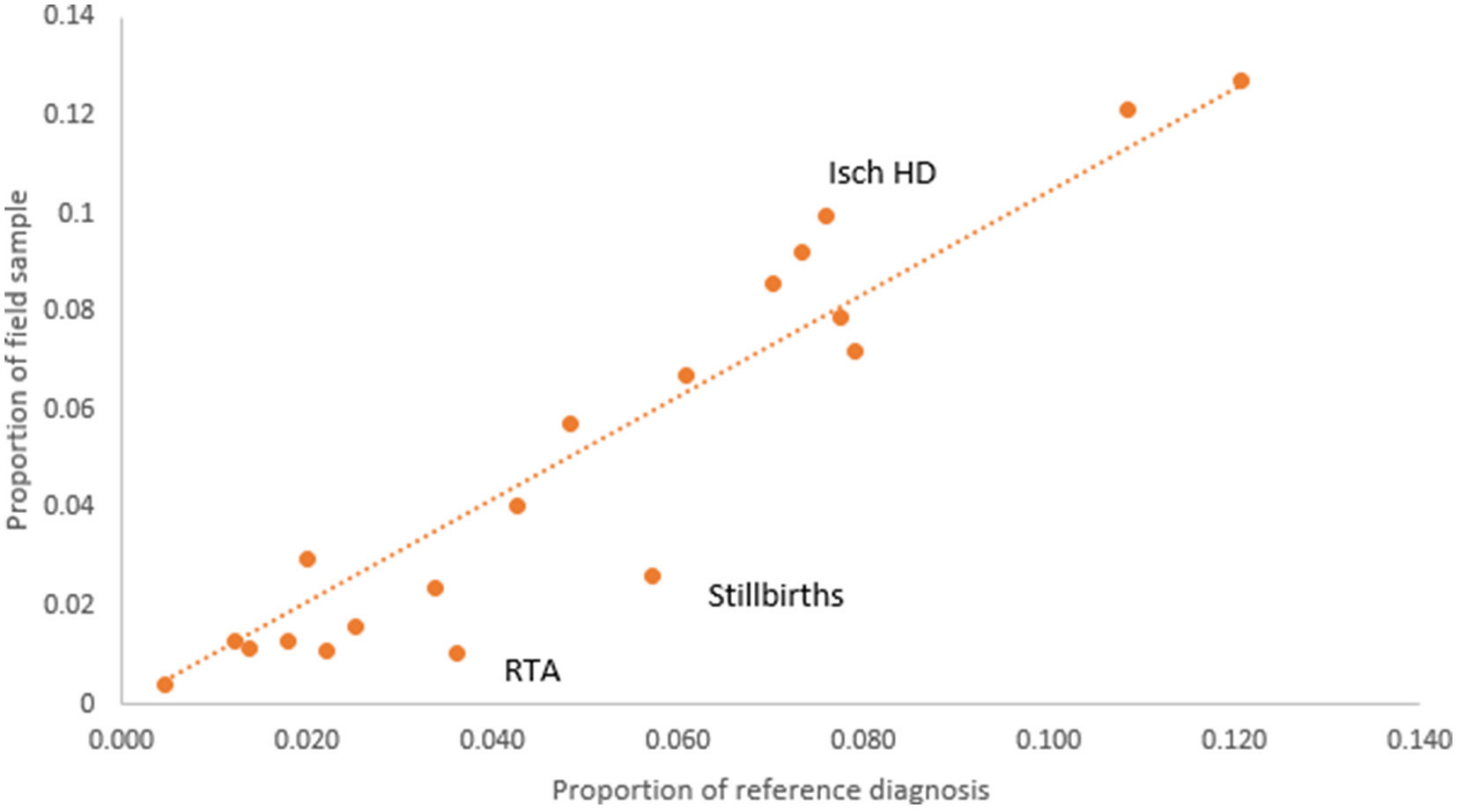
Comparison of COD proportions according to reference diagnoses from the VA target sample (*n* = 7,504) and final VA field sample (*n* = 2,120).

We examined the potential for bias in the study sample from losses to follow-up by comparing the proportionate distribution of reference diagnoses from the target of 7,504 cases with the proportionate distribution of the reference diagnoses for the completed field sample of 2,120 cases (see Figure 2). It can be observed that for the majority of the causes, the losses to follow-up were non-differential except for stillbirths and road traffic accidents (RTA), which were less frequent in the field sample, and ischemic heart diseases (Isch D), which were oversampled. The reason for greater losses to follow-up for stillbirths was because the address was obtained from hospital registers and not clinical case records, which usually contain more detailed and specific information. Our study did not get permission to access stillbirth case records since they were the subject of a concurrent clinical research study and hence could not be shared. For ischemic heart disease, the final study sample did not achieve the target sample of 250 cases, despite achieving higher proportions in the field sample as compared to that in the target sample. Male deaths accounted for 61% of the final field sample of 2,120 cases, of which 15.4% were from stillbirths and neonatal age, and 61% were from the age range of 15–69 years which represents premature adult mortality.

### 3.1. Comparative validation for specific causes

Table 1 shows the proportional distribution of causes of death from the reference diagnoses for the 20 causes of death selected for this study, in comparison with similar distributions as derived from the PCVA and the three CCVA methods. While the reference diagnoses dataset comprises cases entirely from this selected list of 20 causes of interest, each VA COD method assigned some cases to an “undetermined” category, as well as to causes other than the study list. These were relatively higher for the InterVA 5 and InSilico VA programs, with the majority of these deaths classified to unspecified cardiac and infectious diseases. On initial comparison across the VA COD methods, it was observed that only 293 (15.2%) cases were assigned the same COD by all the VA diagnostic methods, this suggests considerable variations in the diagnostic logic applied by the various software programs for assigning causes of death. We assessed these variations by measuring the relative difference in CSMF derived by each method for specific causes from the reference CSMF, also presented in Table 1. Using a threshold relative difference of ±20% to signify acceptable variation (Quigley et al., 1999), we found that PCVA met this threshold for 7 of the 10 leading causes of death, as compared to 3 out of 10 for InterVA5 and InSilicoVA and 2 out of 10 for the Tariff method. Other important findings from the CSMF comparison are that Chronic Obstructive Pulmonary Disease was under-diagnosed by PCVA as well as all the CCVA methods and cardiovascular diseases (stroke and ischemic heart disease) were under-diagnosed by InSilicoVA, and overdiagnosed by the Tariff method. Diabetes was grossly overdiagnosed as an underlying COD by PCVA as well as Tariff.

**Table 1:** Comparison of distributions of underlying causes of death from reference diagnoses and four test COD assignment methods (*n* = 2,120).

		**GS/RS**		**PCVA**			**Inter VA**			***InSilico***			**Tariff**		

**S. No**.	**Disease**	**No**	**CSMF**	**No**	**CSMF**	**RD%**	**No**	**CSMF**	**RD%**	**No**	**CSMF**	**RD%**	**No**	**CSMF**	**RD%**
1	Stroke	277	13.07	201	9.48	−27.44	228	10.76	**−17.65**	157	7.41	−43.32	181	8.54	−34.63
2	Liver disease	260	12.26	217	10.24	**−16.54**	83	3.92	−68.06	112	5.28	−56.92	165	7.79	−36.51
3	Ischemic heart disease	213	10.05	247	11.65	**15.96**	167	7.88	−21.56	119	5.61	−44.13	308	14.54	44.67
4	Cancers	196	9.25	204	9.62	**4.08**	273	12.88	39.35	239	11.27	21.94	126	5.95	−35.68
5	Coronary obstructive pulmonary disease	187	8.82	75	3.54	−59.89	32	1.51	−82.88	7	0.33	−96.26	99	4.67	−47.03
6	Acute respiratory infection	161	7.59	60	2.83	−62.73	170	8.02	**5.64**	260	12.26	61.49	57	2.69	−64.58
7	Prematurity	159	7.50	170	8.02	**6.92**	37	1.75	−76.72	127	5.99	−20.13	124	5.85	−21.98
8	Tuberculosis	140	6.60	137	6.46	**−2.14**	106	5.00	−24.25	88	4.15	−37.14	113	5.33	**−19.25**
9	Kidney disease	118	5.57	119	5.61	**0.85**	75	3.54	−36.41	70	3.30	−40.68	106	5.00	**−10.13**
10	Birth asphyxia	81	3.82	63	2.97	−22.22	49	2.31	−39.48	106	5.00	30.86	13	0.61	−83.94
11	Diabetes	62	2.92	123	5.80	98.39	65	3.07	**4.89**	43	2.03	−30.65	152	7.17	145.28
12	Meningitis	51	2.41	6	0.28	−88.24	17	0.80	−66.65	6	0.28	−88.24	8	0.38	−84.31
13	Stillbirths	40	1.89	42	1.98	**5.00**	30	1.42	−24.96	41	1.93	**2.50**	40	1.89	**0.05**
14	Maternal causes	38	1.79	38	1.79	0.00	60	2.83	57.97	71	3.35	86.84	43	2.03	13.21
15	Neonatal sepsis	32	1.51	6	0.28	−81.25	19	0.90	−40.60	5	0.24	−84.38	9	0.42	−71.86
16	Diarrhea	28	1.32	13	0.61	−53.57	46	2.17	64.36	24	1.13	**−14.29**	29	1.37	**3.62**
17	Accidental fall	24	1.13	38	1.79	58.33	46	2.17	91.76	79	3.73	229.17	59	2.78	145.95
18	Road traffic accident	23	1.08	32	1.51	39.13	35	1.65	52.25	12	0.57	−47.83	35	1.65	52.25
19	HIV	20	0.94	15	0.71	−25.00	19	0.90	**−4.96**	8	0.38	−60.00	0	0.00	−100.00
20	Malaria	10	0.47	4	0.19	−60.00	0	0.00	−100.00	0	0.00	−100.00	0	0.00	−100.00
21	Others	0	0.00	93	4.39	0	406	19.16	0	502	23.68	0	204	9.63	0
22	Undetermined	0	0.00	217	10.24	0	156	7.36	0	44	2.08	0	248	11.70	0

The bold values indicate the relevant findings.

The diagnostic accuracy of each method for individual causes was also assessed using sensitivity and positive predictive value, as shown in Table 2. As can be seen, the PCVA method shows the highest sensitivity scores for 15 out of the 20 causes of death of interest. In comparison, the Tariff methods showed the highest sensitivity scores for three causes, and the InSilico and InterVA methods for one cause each. All VA diagnostic methods scored very low sensitivity scores for all infectious diseases, including neonatal sepsis. The PCVA method showed moderate sensitivities for TB (56%) and HIV/AIDS (53%). It should be noted that all three CCVA programs did not diagnose malaria since the epidemiological parameters of these software programs were set to low prevalence for malaria in India. The Tariff program also assigned low prevalence settings for HIV/AIDS, resulting in zero deaths. While the sensitivity scores for chronic obstructive lung disease were also low from all diagnostic methods, the positive predictive values were reasonably better, indicating that VA diagnosis of COPD tended to be accurate when assigned. However, the PPV scores for diabetes were relatively lower for all methods, indicating an overall propensity for VA to over-diagnose this condition as a COD.

**Table 2:** Cause-specific validation scores of different COD assignment methods for the study sample of 2,120 cases.

	**PCVA**		**Inter-VA**		**InSilico**		**Tarrif**	
**Disease**	**Sensitivity (CI)**	**PPV (CI)**	**Sensitivity (CI)**	**PPV (CI)**	**Sensitivity (CI)**	**PPV (CI)**	**Sensitivity (CI)**	**PPV (CI)**
Stroke	**60 (53, 67)**	83 (78, 88)	44 (37, 50)	53 (47, 59)	35 (28, 43)	63 (55, 70)	41 (34, 48)	63 (56, 70)
Liver disease	**66 (60, 73)**	80 (74, 85)	19 (11, 28)	61 (50, 71)	29 (20, 37)	67 (59, 76)	**50 (42, 58)**	79 (73, 85)
Ischemic heart disease	**66 (60, 72)**	57 (51, 63)	35 (27, 42)	44 (37, 52)	25 (17, 33)	45 (36, 54)	**72 (67, 77)**	50 (44, 55)
Cancers	**81 (76, 86)**	78 (72, 84)	**51 (45, 56)**	36 (30, 42)	**57 (50, 63)**	46 (40, 53)	46 (37, 55)	72 (64, 80)
COPD	31(20, 41)	77 (67, 86)	11 (0, 22)	65 (49, 82)	3 (0, 16)	85 (59, 100)	34 (25, 44)	65 (56, 75)
Tuberculosis	49(40, 57)	50 (41, 58)	30 (21, 39)	40 (31, 49)	25 (16, 34)	40 (30, 51)	37 (28, 46)	46 (36, 55)
Acute respiratory infection	22 (11, 32)	60 (47, 72)	21 (14, 27)	20 (13, 26)	31 (26, 37)	19 (14, 24)	21 (10, 31)	59 (46, 72)
Diabetes	**53 (44, 62)**	26 (18, 34)	19 (9, 28)	18 (9, 27)	16 (5, 27)	23 (10, 35)	46 (38, 54)	19 (12, 25)
Kidney disease	**50 (41, 59)**	50 (41, 59)	23 (14, 33)	37 (26, 48)	23 (13, 33)	40 (28, 51)	35 (26, 44)	39 (30, 48)
HIV/AIDS	**50 (24,75)**	66 (42, 90)	20 (2, 37)	21 (2, 39)	20 (0, 47)	50 (15, 84)	–	–
Diarrhea	10 (0, 27)	23 (0, 45)	28 (15, 41)	17 (6, 28)	10 (0, 23)	12 (0, 25)	25 (9, 40)	24 (8, 39)
Prematurity	83 (77, 88)	77(71, 83)	12 (1, 23)	54 (37, 70)	49 (40, 57)	61 (52, 69)	**59 (50, 67)**	75 (68, 83)
Birth asphyxia	56 (44, 69)	73 (62, 83)	23 (11, 35)	38 (25, 52)	**53 (43, 62)**	40 (31, 49)	6 (0, 19)	38 (12, 64)
Neonatal sepsis	12 (0, 38)	66 (28, 100)	3 (0, 10)	5 (0, 15)	0 (0, 0)	0 (0, 0)	12 (0, 34)	44 (11, 76)
Stillbirths	85 (74, 95)	80 (69, 92)	**67 (50, 84)**	89 (79, 100)	**80 (67, 92)**	78 (65, 90)	**77 (64, 90)**	77 (64, 90)
Malaria	10 (0, 39)	25 (0, 67)	–	–	–	–	–	–
Meningitis	7 (0, 29)	66 (28, 100)	18 (0, 36)	52 (29, 76)	9 (0, 33)	83 (53, 100)	7 (0, 26)	49 (15, 84)
Maternal causes	94 (87,100)	94 (87, 100)	**84 (74, 93)**	53 (40, 65)	**92 (85, 98)**	49 (37, 60)	**78 (66, 91)**	69 (56, 83)
Road traffic accidents	95 (88,100)	68 (52, 84)	**65 (49, 80)**	42 (26, 59)	43 (15, 71)	83 (62, 100)	**95 (88, 100)**	62 (46, 78)
Accidental falls	75 (61, 88)	47 (31, 63)	**50 (35, 64)**	26 (13, 38)	**70 (60, 80)**	21 (12, 30)	**75 (63, 86)**	30 (18, 42)
Others	–	–	–	–	–	–	–	–
Undeermined	–	–	–	–	–	–	–	–

The bold values indicate the relevant findings.

The sensitivity scores for maternal causes of death are relatively high for all methods but are highest at 95 (88–100) for PCVA. In regard to perinatal causes of death, however, the PCVA alone showed high scores of sensitivity for both stillbirths and prematurity and a moderate score for birth asphyxia. In contrast, the CCVA methods did not perform adequately for perinatal causes of death. Finally, for deaths from road traffic accidents, two of the CCVA methods—InterVA5 and InSilicoVA showed low validation scores, although these deaths are relatively straightforward to identify through VA. Similarly, for deaths from falls, all methods showed poor positive predictive values which is indicative of over-identification of this cause by VA.

The diagnostic accuracy of each method can also be understood from the misclassification patterns between the method being tested and the reference diagnoses for the study sample, as presented in Tables 3–6. In each of these tables, the figures in bold font represent the number of cases for which there is a positive agreement in diagnosis from the test method as well as the reference diagnosis. As can be seen, there is a common pattern of misclassification of deaths across major non-communicable diseases, including cardiovascular disease (ischemic heart disease and stroke), cancers, COPD, liver diseases, kidney diseases, and diabetes. There are often common symptoms and overlapping clinical presentations for these causes, which can make it difficult even for physician reviewers to correctly identify the underlying cause in some instances. There are also similar patterns of misclassification from all methods between the closely related perinatal causes of death, including stillbirths, prematurity, birth asphyxia and sepsis. Also, the InterVA method did not assign stillbirth as a cause for any of the cases in the sample, which were hence misclassified to the “undetermined” category. However, it can also be seen that the InterVA5 method also misclassified a considerable number of cases of prematurity and birth asphyxia to the “undetermined” category.

**Table 3:** Misclassification of PCVA diagnoses when compared to reference diagnoses for the study sample.

**Reference diagnosis**	**PCVA**																						
**Diseases**	**ARI**	**AF**	**BA**	**CAN**	**COPD**	**DM**	**DIR**	**HIV**	**IHD**	**KD**	**LD**	**MR**	**MT**	**MN**	**NS**	**OT**	**PM**	**RTA**	**SB**	**ST**	**TB**	**UD**	**Grand total**
Acute respiratory infections (ARI)	**36**	1		2	3	19	1	1	18	11	1	1				16	3			3	16	29	161
Accidental fall (AF)		**18**																1		2		3	24
Birth asphyxia (BA)	1		**46**												1	2	20		4			7	81
Cancers (CAN)	1			**160**		2			5	3	6					3				2	4	10	196
COPD	7	2		5	**58**	13	3	2	31	10	8					8				5	14	21	187
Diabetes (DM)				3		**33**			3	5						1		2			3	12	62
Diarrhea (DIR)		2			1	5	**3**		3	1	4					5					1	3	28
HIV/AIDS (HIV)	1						1	**10**		2	3									1	1	1	20
Ischemic heart disease (IHD)	3			4	6	16			**142**	3	1					5				7		22	213
Kidney diseases (KD)		1		2	1	11	1		7	**60**	7					7				2	4	15	118
Liver diseases (LD)		1		8	1	4	1	1	10	12	**174**					11		3		4	11	19	260
Malaria (MR)										1		**1**	1			4						3	10
Maternal causes (MT)					1				1				**36**										38
Meningitis (MN)		1		2		4	1	1	3	2	1	1	1	**4**		11	1			3	4	11	51
Neonatal sepsis (NS)	3		6												**4**	1	11		2			5	32
Prematurity (PM)	1		0												1	4	**132**		2			9	159
Road traffic accidents (RTA)									1									**22**					23
Stillbirths (SB)			1													1	3		**34**			1	40
Stroke (ST)	2	1		10	1	11	1		20	3	3					11		3		**168**	7	26	277
Tuberculosis (TB)	5			8	3	5	1		3	6	9	1		2		3		1		4	**69**	20	140
**Grand total**	**60**	**38**	**63**	**204**	**75**	**123**	**13**	**15**	**247**	**119**	**217**	**4**	**38**	**6**	**6**	**93**	**170**	**32**	**42**	**201**	**137**	**217**	**2,120**

The bold values indicate the relevant findings.

**Table 4:** Misclassification of InterVA5 diagnoses when compared to reference diagnoses for the study sample (*n* = 2,120).

**Reference diagnosis**	**Inter-VA diagnoses**																					
**Diseases**	**ARI**	**AF**	**BA**	**CAN**	**COPD**	**DM**	**DIR**	**HIV**	**IHD**	**KD**	**LD**	**MT**	**MN**	**NS**	**OT**	**PM**	**RTA**	**SB**	**Stroke**	**TB**	**UD**	**Grand total**
Acute respiratory infection (ARI)	**34**	1		4	1	4	6	2	5	9	3	1	2		39	1			10	10	29	161
Accidental fall (AF)	1	**12**													4		4		3			24
Birth asphyxia (BA)	1		**19**											5	20	11		3			22	81
Cancers (CAN)	9	2		**100**	1	3	4	3	4	3	8	4			24		2		11	7	11	196
COPD	39	2		18	**21**	2	2	1	15	6	3				52				9	17		187
Diabetes (DM)	5			4		**12**	1	1	3	4	3	2	1		20		1		3	1	1	62
Diarrhea (DIR)		1		1	1	2	**8**		2	1	1	2	1		5				1	1	1	28
HIV/AIDS (HIV)		1		4		1	1	**4**	1						3				2	3		20
Ischemic heart disease (IHD)	40	2		8	1	11	1		**75**	6	6	1			44		1		13	2	2	213
Kidney diseases (KD)	6			10		9	5	3	10	**28**	2	2			23		1		13	5	1	118
Liver diseases (LD)	4			93	2	4	10	2	10	10	**51**	4	1		45		4		14	3	3	260
Malaria (MR)												**1**	1		4				4			10
Maternal causes (MT)					1		1					**32**			3				1			38
Meningitis (MN)	3	2		2		3	1		1	2		3	**9**		11				4	3	7	51
Neonatal sepsis (NS)			5											**1**	10	5					11	32
Prematurity (PM)	1		23										1	13	50	**20**					51	159
Road traffic accidents (RTA)		5													3		**15**					23
Stillbirths (SB)			2												3			**27**			8	40
Stroke (ST)	14	18		10	4	9	2	2	39	5	3	4	1		24		6		**122**	11	3	277
Tuberculosis (TB)	13			19		5	4	1	2	1	3	4			19		1		18	**43**	7	140
**Grand total**	**170**	**46**	**49**	**273**	**32**	**65**	**46**	**19**	**167**	**75**	**83**	**60**	**17**	**19**	**406**	**37**	**35**	**30**	**228**	**106**	**157**	**2,120**

The bold values indicate the relevant findings.

**Table 5:** Misclassification of InSilicoVA diagnoses when compared to reference diagnoses for the study sample (*n* = 2120).

**Reference diagnosis**	**InSilico diagnoses**																					
**Diseases**	**ARI**	**AF**	**BA**	**CAN**	**COPD**	**DM**	**DIR**	**HIV**	**IHD**	**KD**	**LD**	**MT**	**MN**	**NS**	**OT**	**PM**	**RTA**	**SB**	**ST**	**TB**	**UD**	**Grand total**
Acute respiratory infection (ARI)	**51**	2		2		1	2		3	6	3	1			54	4			4	12	16	161
Accidental fall (AF)	2	**17**													3				2			24
Birth asphyxia (BA)	2		**43**											2	4	26		4				81
Cancers (CAN)	7	2		**112**		1	1		3	2	7	5			39				6	3	8	196
COPD	56	2		23	**6**	2			10	6	4				57				8	13		187
Diabetes (DM)	7	2		4		**10**	1	1	3	4	2	3			23				1		1	62
Diarrhea (DIR)	2	1					**3**		1		1	1	1		17					1		28
HIV/AIDS (HIV)		1		2		1		**4**	1	1	1				7	1				1		20
Ischemic heart disease (IHD)	58	2		5		5			**54**	6	9	1			65				6	2		213
Kidney diseases (KD)	8			9		8	8	2	7	**28**	3	5			25				10	4	1	118
Liver diseases (LD)	8	5		53		1	5		5	9	**76**	4			78		1		6	5	4	260
Malaria (MR)												**3**			7							10
Maternal causes (MT)	1											**35**			1				1			38
Meningitis (MN)	4	5					1		1	2		2	**5**		20	1			4	1	5	51
Neonatal sepsis (NS)	1		10												7	11		2			1	32
Prematurity (PM)	10	1	52											3	12	**78**		3				159
Road traffic accidents (RTA)		11													2		**10**					23
Stillbirths (SB)			1													6		**32**			1	40
Stroke (ST)	25	27		6	1	11			29	5	3	6			54		1		**99**	10		277
Tuberculosis (TB)	18	1		23		3	3	1	2	1	3	5			27				10	**36**	7	140
**Grand total**	**260**	**79**	**106**	**239**	**7**	**43**	**24**	**8**	**119**	**70**	**112**	**71**	**6**	**5**	**502**	**127**	**12**	**41**	**157**	**88**	**44**	**2,120**

The bold values indicate the relevant findings.

**Table 6:** Misclassification of Tariff 2.0 diagnoses when compared to reference diagnoses for the study sample (*n* = 2,120).

**Reference diagnosis**	**Tariff diagnoses**																				
**Diseases**	**ARI**	**AF**	**BA**	**CAN**	**COPD**	**DM**	**DIR**	**IHD**	**KD**	**LD**	**MR**	**MN**	**NS**	**OT**	**PM**	**RTA**	**SB**	**Stroke**	**TB**	**UD**	**Grand total**
Acute respiratory infection (ARI)	**34**	4		3	4	14	2	20	6		2	1		20	1		1	5	13	31	161
Accidental fall (AF)		**18**														2		1		3	24
Birth asphyxia (BA)	1		**5**										2	17	22		3			31	81
Cancers (CAN)	2	1		**91**	4	13	5	11	8	11				11		1	1	8	14	15	196
COPD	5	2		4	**65**	13	1	40	9	6	1			4				7	13	17	187
Diabetes (DM)				1	1	**29**	2	9						6		2		3	3	6	62
Diarrhea (DIR)		2		1		1	**7**	4	1	1	1			2				1	1	6	28
HIV/AIDS (HIV)		1			1				2	2	1			4			1	3	1	4	20
Ischemic heart disease (IHD)	3	2		1	7	7	1	**154**	3	3	1			7				13	2	9	213
Kidney diseases (KD)	1			1	4	26	2	11	**42**	3	2			10				6	4	6	118
Liver diseases (LD)		2		3	2	18		13	17	**131**	2			34		4		8	5	21	260
Malaria (MR)								1	2		**1**			1						5	10
Maternal causes (MT)					1			4	1		30			1						1	38
Meningitis (MN)		5		4		4	4	1				**4**		10	1			5		13	51
Neonatal sepsis (NS)												1	**4**	13	6		2			6	32
Prematurity (PM)	5		3										3	29	**94**		1			24	159
Road traffic accidents (RTA)								1								**22**					23
Stillbirths (SB)			5											2			**31**			2	40
Stroke (ST)	2	22		11	5	19	1	32	6	1				22		3		**115**	5	33	277
Tuberculosis (TB)	4			6	5	8	4	7	9	7	2	2		11		1		6	**52**	16	140
**Grand total**	**57**	**59**	**13**	**126**	**99**	**152**	**29**	**308**	**106**	**165**	**43**	**8**	**9**	**204**	**124**	**35**	**40**	**181**	**113**	**249**	**2,120**

The bold values indicate the relevant findings.

### 3.2. Indicators of the overall accuracy of the COD assignment method

The indicators for the overall comparison of the performance of each COD assignment method are presented in Table 7. The PCVA method achieved the highest accuracy out of all methods for all three metrics used for this assessment, with considerable margins of difference. Of these indicators, the scores of positive agreements can be interpreted readily in conjunction with evidence from Tables 3–6. However, the CSMF Accuracy is more complex and can be influenced by compensatory misclassification patterns that could minimize the net effect of differences in cause proportions from a test method with those from the reference standards. While this study shows consistency in the performance of methods across all these summary metrics, they should be utilized in conjunction with the comparative findings of accuracy measures for individual causes of death for overall adjudication on the ranking of methods for diagnostic performance.

**Table 7:** Metrics for overall accuracy of COD assignment methods (2,120).

**Methods**	**% agreement**	**Kappa**	**CSMF accuracy**
PCVA	57.10	0.54	0.79
Inter-VA5	29.86	0.26	0.66
InSilicoVA	32.97	0.29	0.62
Tariff	43.77	0.40	0.67

## 4. Discussion

With the advent of computerized methods for assigning causes of death from VA, this study provides important evidence of the performance of these methods in the Indian context. The principal finding that PCVA appears to be the most accurate COD assignment method from VA data is not unexpected, particularly since physician reviewers have access to and can wholly utilize information from the open narrative section of the VA questionnaires, which has been proved from previous research to provide valuable and accurate evidence to establish the probable COD. Nevertheless, the quantification of computer diagnostic performance using well-defined reference diagnoses as well as standard statistical indicators for performance evaluation, along with the detailed symptom-cause information compiled from this study, will be a valuable resource for further development of the diagnostic logic of the CCVA programs. In parallel, the diagnostic errors noted for PCVA in Table 3 can also be evaluated with detailed information from questionnaires and guide training programs to strengthen the implementation of PCVA for COD assignment in India.

Validation of verbal autopsy diagnosis is challenging in terms of obtaining reference diagnosis. Several studies have used causes of death based on medical records as the reference standard (Byass et al., 2003; Bauni et al., 2011; Leitao et al., 2014; McCormick et al., 2016; Nichols et al., 2018; Samuel and Clark, 2018). However, these could be limited by the quality of evidence from medical records for establishing reference diagnoses. Our study established reference standards from medical records for deaths in five tertiary care teaching hospitals in Delhi. These diagnoses were formulated by senior resident physicians who were rigorously trained in medical certification of COD and essential principles for assigning the underlying COD. Further, these physicians were supervised in their work by departmental teaching staff in each hospital and also the central study team, which played a major role in selecting medical records with the highest quality of diagnostic evidence for the study. In addition, our study adopted the strategy of taking matched pairs of underlying causes from two independent physician reviews of hospital case files and this ensured consistency in the application of ICD rules for the selection of underlying COD. We also used the same approach for deriving underlying causes from VA, in which we found primary agreement on the underlying cause for 65% of cases between two independent PCVA reviewers, to which an additional 26% were added based on an agreement between either of the mismatched diagnoses and the cause assigned from the third review. These findings underscore the rigor in study procedures used for assigning reference standards and PCVA diagnoses.

A review of studies that compare the performance of CCVA and PCVA methods published in 2014 observed that the Tariff method measured the key performance indicator of CSMF Accuracy to be 0.71 and the PCVA method to be 0.68 (Leitao et al., 2014). Similarly, another analysis by Murray et al. reported that the CSMF accuracy from Tariff was in the range from 0.76 to 0.81 across different age groups, while in comparison, the PCVA method had a CSMF Accuracy that ranged from 0.68 to 0.71 (Murray et al., 2014). Both these analyses were based on data collected from an international field study conducted during 2008–2009 in Tanzania, Philippines, Mexico, and India. In contrast, our study observed that physician certification of VA consistently achieved the higher scores of all methods across the entire range of indicators used to evaluate validity and accuracy at the level of individual causes presented in Tables 1–6 as well as for the measures of overall performance in Table 7.

Studies have used machine learning approaches in addition to CCVA algorithms to improve upon the accuracy of automated methods for assigning causes of death. Studies have shown that analyzing narratives can enhance machine learning model prediction if they are added to the responses of structured questionnaire (Reeves and Quigley, 1997; Mujtaba et al., 2019; Mapundu et al., 2022). AI models, such as deep learning neural networks and machine learning algorithms, have been trialed to process large volumes of verbal autopsy data and extract meaningful patterns. For instance, studies have attempted to utilize convolutional neural networks (CNNs) to automatically classify verbal autopsy narratives into specific cause-of-death categories (Murtaza et al., 2018). Natural language processing (NLP) techniques, combined with AI models, have also enabled the extraction of key features, linguistic cues and sentiment analysis from verbal autopsy texts, providing insights into the emotional context surrounding a death (Danso et al., 2013).

However, the PCVA method does show low levels of accuracy for some conditions, which needs explanation as well as attention for improvement. For instance, a considerable number of stroke deaths (as per reference diagnoses) have been misclassified to accidental falls. This could be simply because the family member could only recall and report the fall associated with the sudden unconsciousness that occurs from an acute stroke without having the opportunity to observe unilateral paralysis, which is the cardinal sign of stroke. Similarly, there are specific patterns of misclassification in regard to the COD assignment for deceased individuals with diabetes. Some physicians nominate diabetes as the underlying cause when present along with ischaemic heart disease and stroke, while others consider such co-morbidity as an association rather than a causal relationship and hence nominate the cardiovascular condition as the underlying cause. The ICD rules permit physicians to exercise their individual preferences for these situations, which therefore explains the observed cross-classification patterns observed from PCVA. However, it is interesting to note similar cross-classification patterns between diabetes and cardiovascular diseases even from CCVA methods in Tables 4–6, where a consistent pattern would have been the expectation. At least for PCVA, there is a need for recommending one standard practice in underlying cause selection for such instances, or allowing for reporting multiple causes of death, in COD, which could enable more in-depth analysis of data quality as well as provide additional epidemiological evidence on co-morbidities at death. However, more attention is required to standardize PCVA diagnostic and certification practices, particularly in improving consistency in assigning deaths due to diabetes. Also, qualitative research with the group of physicians involved in PCVA could also help understand the challenges in performing their tasks, in terms of ambiguity in the VA responses, the potential to modify the questionnaire, improvement in the quality of recording of open narratives, and other aspects concerning training of VA interviewers.

A potential limitation of the study was the relatively large number of cases (74%) that were lost to follow-up by VA, arising from incomplete or wrong address details provided on case records. Field supervisors made follow-up visits to search for houses not identified by VA interviewers in about 10% of cases, but these visits, too, were largely unsuccessful, hence confirming the loss to follow-up. However, as demonstrated in Figure 1, these losses were largely non-differential by cause, which minimizes the potential for bias from this aspect. These findings prompt the need for greater attention to recording identity and address details on hospital clinical records.

For assigning causes of death, we selected a set of 20 causes of death that we considered important for the epidemiological profile of India. However, our target list did not include other less frequent but still important conditions such as dengue fever, leishmaniasis, and hypertensive/congestive heart disease. Validation of VA diagnoses for these conditions would require specially designed studies that separately target these conditions, given their seasonality or geographical specificity. Nevertheless, the conduct of this study has established a standard methodology for VA validation studies in India, which could be replicated in other settings with varying epidemiological profiles across India.

## 5. Conclusion

In conclusion, our study found that the PCVA method had the best performance out of all the four COD assignment methods that were tested in our study sample. While the CCVA algorithms have not yet achieved an appropriate level of accuracy for them to be directly taken as alternatives to physician assignment of causes of death, the data compiled from studies such as this one could be very useful for improving both PCVA and CCVA programs.

## Data availability statement

The datasets presented in this article are not readily available because, the datasets generated and/or analyzed during the current study are not publicly available, as we are a Government of India research organization; approvals from the competent authority need to be taken for data sharing, but the data may be available from the corresponding author on reasonable request, subject to approval from the competent authority. Requests to access the datasets should be directed to MR, dr_vishnurao@yahoo.com.

## Ethics statement

The studies involving human participants were reviewed and approved by ICMR-NIMS Ethics Committee on Health Research. The patients/participants provided their written informed consent to participate in this study.

## Author contributions

MR, SB, SS, and CR conceived and designed the analysis and wrote the paper. MR, SB, SS, SN, AJ, LS, and BG conduct of study. SN, KS, SS, LS, SN, AJ, VY, and BG contributed to data analysis and interpretation. MR, KS, LS, and AJ performed the analysis. All authors contributed to the article and approved the submitted version.
