# Erratum: Evaluation of methods for assigning causes of death from verbal autopsies in India

**DOI:** 10.3389/fdata.2023.1319729

**Published:** 2023-10-24

**Authors:** Frontiers Production Office

**Affiliations:** Frontiers Media SA, Lausanne, Switzerland

**Keywords:** cause of death, computer-coded verbal autopsy (CCVA), physician-coded verbal autopsy (PCVA), verbal autopsy, India

Due to a production error, there was an error in the affiliations for authors Ved Prakash Yadav and Chalapati Rao. Instead of author Ved Prakash Yadav having affiliation with “College of Health and Medicine, Australian National University, Canberra, ACT, Australia”, they should have “World Health Organization, New Delhi, India”. Likewise for author Chalapati Rao, instead of having affiliation with “World Health Organization, New Delhi, India”, they should have “College of Health and Medicine, Australian National University, Canberra, ACT, Australia”. The publisher apologizes for this mistake. The original article has been updated.

